# Freire’s view of a progressive and humanistic education: Implications for medical education

**DOI:** 10.15694/mep.2017.000119

**Published:** 2017-07-03

**Authors:** Dario Torre, Victoria Groce, Richard Gunderman, John Kanter, Steven Durning, Steven Kanter

**Affiliations:** 1 Uniformed Services University of the Health Sciences; 2 University of Pittsburgh School of Medicine; 3 Indiana University School of Medicine; 4 Dartmouth-Hitchcock Health System; 5 University of Missouri-Kansas City School of Medicine

**Keywords:** medical education, educational philosophy, educational theory

## Abstract

This article was migrated. The article was marked as recommended.

Brazilian educator Paulo Freire argues that the purpose of education is to liberate human potential and, thus, is much more than a teacher simply depositing information into the mind of a learner. His ideas are important to medical education because 1) they strengthen the philosophical underpinning of practices in medical education (for example, Freire’s dialogical approach to learning vis-à-vis the banking model of education adds philosophic strength to the use of problem-based learning as a primary learning modality); 2) they encourage medical educators to confront directly the tension between teaching for conformity, which many would argue is key to performing a good and reliable physical examination, and teaching to liberate human potential, which is critical to preparing physicians to question current assumptions, practices, and knowledge; and 3) they encourage expanded thought about approaches to medical education, for example in the choice of pedagogical method, defining the role of the educator, and the use of experiences like service learning.

## Introduction

Paulo Freire (1921-1997) was one of the most influential educational theorists of the latter half of the 20th century and his ideas continue to influence thinking today. He was a Brazilian educator, scholar, and social theorist who conceived a humanistic philosophy of values that considers knowledge to be a product of human practices that can transform the world. He developed a successful literacy program in Latin America and Africa, and his work has influenced many fields including pedagogy, philosophy, medicine, social sciences, and literature. He fought his entire life to give people access to an education that would empower them to grow and develop to their full capacities.

Freire’s writings are frequently associated with the cause of popular education, such as adult literacy programs, as well as issues such as economic inequality, race, and social justice. [ Bergsma 2004, Choules 2007, Jackson 2008] However, Freire’s perspective also has the potential to illuminate higher education, including medical education. In his most well-known book, *Pedagogy of the Oppressed* [ Freire, 1970], Freire argues that the purpose of education at every level is far more than the transmission of information, a perspective that might surprise many medical students who feel they are being buried under an information avalanche. Instead Freire argues that education is about the liberation of human potential. This article explores Freire’s ideas about education, focusing especially on insights that can inform contemporary medical education.

## Freire’s educational framework

For Freire, education is part of the broader process of liberation: oppressed peoples’ throwing off the yokes of their oppression and replacing dehumanizing social systems with ones that recognize and support the humanity of each individual. Freire argues that most contemporary educational approaches are fundamentally oppressive, in the sense that they constrain freedom. In Freire’s view, the progressive teacher is one who is an agent in the production of knowledge, whose goal is not to transfer the teacher’s superior knowledge to students, but rather to foster a student’s capability for learning and to facilitate the educational process in a way that makes the teacher a fellow learner alongside the student.

Freire’s path to liberation is through raised “conscientização” (critical consciousness) on the part of the oppressed: becoming aware of the situation of oppression and determining how it might be changed without perpetuating an unjust system. In Freire’s conception of history, every era and place has its own major problems. People cannot help interacting with these themes. Freire suggests a series of concentric circles with universal themes of a given era on the outermost circle, themes of broad geopolitical importance on the next circle, and themes of national importance on the next circle. Smaller circles are important themes for ever narrower subcultures within a nation or society. Importantly, while broad themes affect everyone, people often only perceive the narrow themes relevant to their day-to-day lives.

Structuring human problems in terms of these major themes suggests a role for teachers: to help students clarify their understanding of the forces affecting their lives, to help students clarify their thinking in a quasi-Socratic manner, and to help tease out links between themes that arise during dialogue. Freire uses dialogue [ Freire, 1985] as the primary tool here: teachers listen to students to see what problems they are most concerned with, use those themes to pose relevant problems, and help students raise consciousness by considering why given problems exist, what are the broader forces at work, and what solutions could they implement in their communities. Because the problems teachers pose are drawn from the students’ own concerns, they are highly relevant to students and should be inspiring.

Freire contrasts his dialogical approach with the “banking concept of education” in which “knowledge is a gift bestowed by those who consider themselves knowledgeable upon those whom they consider to know nothing.” [ Freire, 1970, p.72]

Freire describes the traditional teacher-student relationship as a non-hierarchical partnership having a narrative character, in which the teacher assumes the role of narrating subject material to which the students listen. Freire states that this creates a form of education that is “lifeless and petrified” and that education suffers from “narration sickness” [ Freire, 1970, p.71]. He writes that as teachers “fill” learners with narrated content, the students memorize the content without critical or transformative thought. As Freire sees it, knowledge is deposited into the students’ minds, creating the banking metaphor of education “in which the scope of action allowed to the students extends only as far as receiving, filing, storing the deposits” [ Freire, 1970, p.72]. Such a concept implies that students are containers, filled with information by the teacher, whose effectiveness is measured by how well he or she can fill such containers. This approach does not motivate students to use their curiosity, does not foster the development of critical thinking skills, and impedes dialogue which is essential for the construction of meaning.

In contrast to the traditional teacher-student relationship, Freire believes that dialogue [Rule 2011] can lead to the development of a different and more effective relationship between educator and learner. In a dialogical model, students and teacher form a partnership and become jointly responsible for learning [ Gadotti 2009]. In this framework, the educator continually rearticulates his or her reflections based on the reflections of students. The students are continually challenged by problems posed by the teacher, become critical co-investigators instead of passive listeners, and become fully engaged through dialogue with the teacher in creative and critical thinking about real problems.

## Considerations in viewing medical education through a Freirean lens

In exploring Freire’s ideas in the context of medical education, one must acknowledge the socio-historical context that informs *Pedagogy of the Oppressed.* In the circumstance in which Freire developed and refined his educational theories, the people for whom he was organizing were being oppressed in systematic, obvious, and clearly dehumanizing ways. It is reasonable to question how much we can separate Freire’s ideas from their original context and whether it is possible to incorporate Freire’s philosophies into modern medical education without dismantling the structures and methods that have evolved over the last century and beyond.

For effective and safe patient care, medical educators require medical students to master a large body of factual material and a significant number of technical skills, and assure they have done so through observation and assessment. This would seem to be at odds with an educational approach where a teacher’s role is to support the student’s own explorations without imposing a top-down curriculum.

While it is not a major focus of *Pedagogy of the Oppressed*, Freire himself notes the potential contradiction here when dealing with bodies of knowledge that involve technical knowledge or craft. But, Freire notes that educating students to enter professions requires a deep understanding of the cultural forces surrounding those professions and a commitment to supporting and recognizing the humanity of all people.

A Freirean lens can stimulate medical educators to look for places where power disparities and dehumanization might compromise the mission of medical education and can help medical educators find ways to rectify those imbalances. Some of the broadest goals of Freire’s approach - i.e., to inspire students to take greater control of the material they are learning and to build positive social structures - are deeply relevant to medical education.

## Implications of Freire’s ideas for medical education

Freire’s ideas are important to medical education because 1) they strengthen the philosophical underpinning of practices in medical education (for example, Freire’s dialogical approach to learning vis-à-vis the banking model of education adds philosophic strength to the use of problem-based learning as a primary learning modality); 2) they encourage medical educators to confront directly the tension between teaching for conformity, which many would argue is key to performing a good and reliable physical examination, and teaching for freedom (or teaching for exploration), which is critical to preparing physicians to question current assumptions, practices, and knowledge; and 3) they encourage expanded thought about our approaches to medical education, including teaching professionalism, the role of the humanities in medical education, and the use of assessment in medical education.

In this article, we will discuss Freire’s ideas in the context of pedagogical methods in medical education, the role of the medical educator, and community health and service learning.

### Pedagogical Methods in Medical Education

In the banking concept of education, the teacher teaches and the students are taught. This is the impression a casual observer would likely glean from many medical school lectures, where a faculty member speaks from the front of the room, while students sit quietly taking notes. It appears that knowledge is being transferred in one direction: from the teacher, who knows, to the students, who are there to learn. Most key decisions appear to be the domain of the teacher, who generates the curriculum, selects the mode of instruction, and devises the assessment of what has been learned. The students’ missions are to listen attentively, memorize as much as they can, and be prepared to reproduce as much as possible on an examination. Students perform well to the extent that their store of knowledge approximates that of the teacher.

Of course, Freire’s criticisms do not apply across the board at all institutions. Some schools, courses, and instructors show few traces of the banking model. And yet, the temptation for educators to adopt a banking approach, particularly in an era of resource constraints, is ever present.

There is an inherent tension between Freire’s ideas about teachers relating to students as “equals” and the fact that medical teachers know more than their students in practical, quantifiable ways. Dialogical learning is likely not an appropriate way to teach a student to insert a chest tube or to suture an incision. But recognizing places where power disparities between student and teacher can be diminished or eliminated may be one way of honoring a student’s humanity.

### The role of the medical educator

According to Freire, the role of the educator is to provide leadership during the problem-posing process, facilitate discussion, and provide support when student participation wanes. Educators should contribute information after group reflections and enter group dialogue, helping learners to develop a critical consciousness of the issues. The educator’s goal is to empower learners and to help create collective knowledge from sharing experiences, ultimately helping learners take their knowledge outside the classroom to apply it in real world settings. For Freire, the dialogical process focuses on reality that, posed as a problem, becomes a challenge to individuals and communities. The role of the educator is not to impose his or her view of the world on the learner, but to establish a dialogue in which their respective views are shared and critically examined. For Freire, the opposite of oppressive education is an educational process that is authentic, liberating, and done with one another instead of “from one to the other.” [ Dos Santos, 2009]. Freire identifies qualities of the progressive educator as humility, the ability to respect others, and the ability to listen to every voice regardless of its intellectual level. [ Freire, 2005] Freire’s description of the role of the educator reinforces the goals of a fully participatory learning experience.

Clearly, Freire’s view of the role of the educator is consistent with the role of the facilitator in problem-based learning [De Grave 1999]. In addition, Freire’s dialogical process can inform the role of attending physician in the clinic or on hospital rounds. It provides a clear philosophic rationale for a teaching physician to abandon traditional quizzing, pimping, and “gotcha” questioning, and instead to encourage learners to develop a critical consciousness of medicine and health care.

### Community Health and Service Learning

Medical schools already recognize the need to develop educational programs that address the health needs of poor and underserved populations. There is evidence, however, that training in community health services could be improved [ Seifert 1998, Drain 2007, Olney 2006]. In the context of community health, medical students are expected to understand the social determinants of health, barriers to accessing care, risk factors and strategies to improve early detection of disease, and cultural issues related to population health [ Kumagai 2009]. Understanding these topics in an academic sense does not necessarily mean that students are learning to empathize with underserved patients. A recent systematic review [ Hunt 2011] about the goals of service learning indicated that such programs often suffer from a lack of collaborative partnership with the community, an unclear process for identifying the community’s needs, and a low frequency of community members as teachers.

Freire’s methods can improve these programs. Because his approach involves a systematic method for eliciting community concerns through dialogue, it can help mitigate “town-gown” conflicts in these programs while giving students a broader and more nuanced view of the lives of the people they serve. And because Freire’s method of learning requires action, a natural consequence of moving toward a more Freirean approach to cultural competency is to allow students to learn with and in the world, deeply understanding key contextual issues that relate to specific health care problems.

Freire’s ideas have significant potential to improve the design and implementation of service-learning curricula [ Elam 2003]. Using Freire’s ideas as a foundation for developing service-learning experiences means immersing learners in the community as fully engaged, curiosity-driven partners who are empowered to think critically as they identify themes through a dialogical methodology [ Heidenmann 2011]. In order to investigate themes and truly learn about the complex interaction of various components that play a role in people’s health, educators and learners need to be fully connected and aware of people’s thinking about their reality. Curricula should create the premises for students and patients to jointly explore health themes as co-investigators. Learners should engage in a process of confronting challenging health issues in a real-life context, discovering relationships among multiple components, and proposing solutions. Only after a critical analysis, working alongside other people, can learners begin to understand how key determinants of health may affect a population, acquiring new skills that will serve them well in the future.

## Discussion

For most of its history, medical education, like all forms of higher education, have been under the influence of what Freire refers to as the banking model of teaching and learning. Even today, in the classroom and maybe more so on the hospital ward, one still can see the influence of the banking model. In the banking model, the pressure for students to keep quiet most of the time runs deep. A student who speaks during a lecture may incur not only the lecturer’s ire but that of fellow students as well. The voice of faculty members is signal, the voice of students is mere noise. Of course, the teacher may pause at some point and ask if the students have questions, though nearly always at the conclusion, when people are feeling overloaded and averse to taking on even more information. And the types of questions students pose make a big difference. Requests for repetition or clarification are not particularly threatening, and teachers are likely to deal with them expeditiously. By contrast, inquiries into basic assumptions, possible inconsistencies, or alternative points of view may receive less sympathetic treatment, and some faculty members may even see them as a waste of valuable time. After all, in the banking model, the student’s mission is not to find a voice, but to listen.

Freire seeks to supplant banking education with another model of pedagogy. From the standpoint of the pedagogy of liberation, learners should be not educational objects but educational subjects. In more recent times, medical educators have made significant progress along these lines, with increased use of active learning in both large- and small-group settings, as exemplified by team-based learning, problem-based learning, and other methods.

The learner’s awareness and active participation are necessary for real education to take place. Rather than submerging learners, the educator’s mission is to bring them to the surface, where they can begin seeing and deciding for themselves. The goal is to help the learner emerge into a fuller consciousness of reality, thereby becoming a more complete human being. If learners - and for that matter, educators - lack freedom to perceive and choose for themselves, we cannot be free, and if we are not free, and thus unable to choose, we cannot be really be human. Quite simply, we are not responsible for what we are compelled to choose. We can only assume responsibility for choices we make ourselves. Freire’s pedagogical model aims to liberate human beings, enabling us to help build a world in which it is easier to work together and care for one another.

Education at its best is a process of discovery, where learners and educators collaborate to develop and test different ways of understanding and acting. Some of these approaches turn out to be insufficiently fruitful. When this happens, we must go back to the drawing board. To regard teachers as pure producers and students as pure consumers is to profoundly misunderstand both. In order for each to function at their best, educators must learn, and learners must educate. Both are co-investigators in a shared inquiry, the object of which is deeper understanding of the world and ourselves. In medicine, the object is a deeper understanding of patients, their medical conditions, and the steps necessary to promote and restore their health. Genuine knowledge is not simply transmissible, but is discoverable, and both educators and learners need to support and encourage one another in this ongoing inquiry. For both, one of the most important resources is the hope that, in spite of obstacles and setbacks, ongoing collaboration will yield new insights.

According to Freire, it is vital that we avoid becoming trapped by a materialistic attitude toward existence, one in which money is the measure of all things. During an era when medical school and university leaders are largely judged according to their ability to attract funding, this warning is particularly timely. Freire simply rejects materialism as the basis of education. In part, he argues that fundamental educational objectives and outcomes, such as professional and personal fulfillment, cannot be commoditized or monetized. More importantly, he argues that being is not having. We are not the objects we possess. Supposing otherwise continually threatens to transform both learners and educators from subjects into objects. By contrast, Freire argues that we are what we know, or at least what we inquire into.

Freire encourages us to be critical, not in the sense of objecting to everything, but in the sense of being committed to lead lives of reflection and dialogue. If learners think their sole mission is to store deposits from teachers, learners will never be inquirers in their own right. Drawing students into dialogue is the teacher’s highest goal, which in turn provides teaching’s highest reward: to help another human being become more fully alive, and in the process become more alive themselves.

It is important to stress that the learners are not the only beneficiaries of Freire’s approach. Moving from education-as-indoctrination to education-as-liberation is no less salutary for educators than learners. For one thing, educators gain the opportunity to regard the “material,” the curriculum, as something alive and transformative, rather than inert and merely transmissible. Contact with learners becomes more engaging and rewarding, because each comes alive when invited to join in the other in dialogue, as opposed to merely speaking or listening to a monologue. Finally, educators discover new vitality in the educational mission. The word education comes from the Latin “to draw out” or “to lead forth.” At its best, education means drawing out of both learners and educators the best of which they are capable. The mission is not to suppress or submerge identities, but to give them full opportunity to express themselves in dialogue.

Entering into such dialogue represents an act of trust. It requires trust in others, that they can become genuine collaborators in the quest for understanding. It requires trust that other people can and will recognize the calling to become more fully human. It also involves humility. We need to be humble enough to admit that we do not have all the answers, and that the inquiry is worth pursuing further. Educators no less than learners need to recognize our own incompleteness [ Freire 1998], that we are not as fully humanly developed as we could be. It is no surprise that the most famous educator of all, Socrates, kept provoking the people of ancient Athens to reexamine what they thought they knew, hoping to awaken in them the recognition that they did not know as much as they thought and had an appetite to know even more. Only people who know that they do not already know everything and who reject the fallacy that those around them are incapable of knowing anything will answer the call to the pedagogy of liberation.

By taking Freire’s perspective to heart, medical education can be powerfully invigorated and, in some cases, transformed [ Wallerstein 1988, Das Gupta 2006, Mooney 2006] Learners cease to be mere receptacles and become legitimate sources of knowledge for educators. Learners become active co-investigators, whose voice is necessary for real conversation to take place. Learners take on moral responsibility for what happens in the classroom, supplanting mere conformity with creativity. Adaptation is no longer a challenge faced only by learners. Instead, learners and educators must adapt to one another, with educators functioning less as drill sergeants and more as partners in the dance of dialogue. To do it well, we must meet our partners, adapt to their movements, and collaborate in creation. Medical education can no longer be something done to learners by educators. Instead, medical education must become a process by learners in partnership with educators, who are enlightened and transformed in the process.

## Notes On Contributors

Dario Torre MD, MPH, PhD is associate professor at the Uniformed Services University of the Health Sciences. He serves as director of the internal medicine clerkship for medical students, residency program director, and director of undergraudate medical education.

Victoria Groce is executive project coordiantor in the Office of the Vice Dean at the University of Pittsburgh School of Medicine.

Richard Gunderman MD, PhD is Chancellor’s Professor of Radiology, Pediatrics, Medical Education, Philosophy, Liberal Arts, Philanthropy, and Medical Humanities and Health Studies at Indiana University.

John Kanter MD is a second-year neurosurgery resident at Dartmouth-Hitchcock Health System.

Steven Durning MD PhD is professor of medicine and pathology at the Uniformed Services University and is the director of graduate programs in health professions education.

Steven Kanter MD is dean of the University of Missouri-Kansas City School of Medicine. He is professor of biomedical and health informatics and holds the Merl and Muriel Hicklin/Missouri Endowed Chair in Medicine.
