# Supplementary material for: Freire’s view of a progressive and humanistic education: Implications for medical education
Source: MedEdPublish (2016). 2017 Jul 3;6:119. [Version 1] doi: 10.15694/mep.2017.000119 (PMC10885273; doi:10.15694/mep.2017.000119)
Supplement: Peer review history

# Reviewer report: Reviewer response for version 1

**DOI:** 10.21956/mep.19305.r28152

**Published:** 2017-07-31

**Authors:** Julie Browne

**Affiliations:** 1 Cardiff University School of Medicine

This review has been migrated. The reviewer awarded 2 stars out of 5 Thank you for this paper: I am glad to see the work of Paolo Freire being brought to the attention of new generations of medical educators and hope that his writing will continue to inspire and enthuse those for whom medical education is a moral, social and transformational enterprise.Paolo Freire is a great writer, passionate and expressive, and it is well worth making the effort to track his books down, particularly Pedagogy of the Oppressed – which was one of the key educational texts that completely revolutionised my thinking about the role of the teacher. It has been built on and discussed many times in the nearly 50 years since it was first published.Which makes this paper a bit of a problem for me. I don’t think it clarifies Freire’s work, and in many respects over-simplifies it. It sets up a ‘straw figure’ – the oppressive lecturer who bullies students into keeping silence – and quite rightly condemns this banking approach to the education of medical students and doctors. But this is actually something of a caricature: and rather dated at that. All over the world medical curricula are changing to become more inclusive, collaborative and socially responsive. New practices and beliefs appear constantly in medical education as the traditional lecture based course is replaced by the latest accepted beliefs (on topics such as technology enhanced learning, competency based education, PBL, and new assessment models). But Freire’s work reminds us constantly that new ideas and practices in education risk becoming unjust and oppressive orthodoxies in themselves if we simply adopt them unquestioningly. He warns that we need to continue to think carefully and reflexively about what we are doing and why we are doing it or we will simply repeat old injustices in new ways. This paper, while clearly passionate and well referenced, takes an overly simplistic approach to Friere’s work in order to criticise an outdated philosophy of teaching that has been significantly superseded in recent years. It then, perhaps a little predictably, advocates replacing these older teaching approaches with a fairly standard appeal for increased partnership and social learning approaches. As a result I think it has missed a great opportunity to move our thinking about medical education on into the future.

Reviewer Expertise:

NA

No decision status is available

# Reviewer report: Reviewer response for version 1

**DOI:** 10.21956/mep.19305.r28149

**Published:** 2017-07-30

**Authors:** Lakshmi Nagappa Naik

**Affiliations:** 1 Melaka Manipal Medical College

This review has been migrated. The reviewer awarded 5 stars out of 5 I truly enjoyed the article and this topic is very close to my heart. The many ideas which flow through this deep exploration of education and its true purpose, has inspired me to come up with teaching modules . Social responsibility, community welfare and health care for all, global inequality in health care have been recognised and being acted upon with apt solutions. However educating our future health care professionals is crucial and especially a role for these in the curriculum is of paramount importance . Community based learning helps in early induction of learners into the realities of health care system in a poignant way ,by experiential learning of a particular community, cultural ethos and disparity in care . I thank the author for this piece of wonderful literature

Reviewer Expertise:

NA

No decision status is available

# Reviewer report: Reviewer response for version 1

**DOI:** 10.21956/mep.19305.r28151

**Published:** 2017-07-13

**Authors:** Trevor Gibbs

**Affiliations:** 1 AMEE

This review has been migrated. The reviewer awarded 2 stars out of 5 I enjoyed reading this article and as an introduction and modest application of Freire's work in education, it is a useful addition to the literature. There must be very few people who could argue against the authors' reflections.However my main concerns as I read the paper were, have we not moved beyond repeating the same conclusions over and over again, should we not be moving forward and looking at how we place these reflections into actions and demonstrating how Freire's words can be applied and evaluating what we hope would happen. Until we stop repeating what we already know such papers as this, in my opinion, lose a lot of their value.

Reviewer Expertise:

NA

No decision status is available

# Reviewer report: Reviewer response for version 1

**DOI:** 10.21956/mep.19305.r28150

**Published:** 2017-07-12

**Authors:** P Ravi Shankar

**Affiliations:** 1 American International Medical University

This review has been migrated. The reviewer awarded 4 stars out of 5 I enjoyed reading this well-written article. Freire’s ideas have a deep relevance to medical education and social justice some of which have been briefly addressed in this article. On a personal note I am of the opinion that Freire’s ideas of justice, social change, and empowerment have serious implications for the healthcare debate raging on in contemporary America. I was first exposed to the ideas of Paulo Freire through the ‘Theater of the oppressed’ developed by the Brazilian theater personality, Augusto Boal who based his ideas on Freire’s work. Theater of the oppressed was fun, interactive and encouraged reflection and change. Having been involved with the Health Humanities for over ten years I sometimes use techniques borrowed from Theater of the oppressed (TO) during the health humanities sessions. Medical education has emphasized information transfer, knowledge retention and reproduction and transformative learning has received very little emphasis. Dialogue is becoming more common in medical education with increasing use of small group learning strategies. However, I believe the traditional scientific knowledge base does constrain and influence generation of knowledge and social and other issues receive less attention. One of the major reasons why lectures remain the dominant teaching modality in many medical schools is its perceived effectiveness in transmitting information to large number of students. In many offshore Caribbean medical schools, lectures remain the predominant teaching strategy. Many faculty members are not introduced to and not in favor of other teaching-learning methods and strategies. In many countries, educational fellowships and programs for medical school faculty are slowly changing the picture. As rightly pointed out community-based learning is becoming increasingly important and the challenge is to make students show greater empathy to the underserved and under-resourced populations. Education and especially medical education is becoming a commodity with students ‘paying’ for an education with the objective of performing well in their examinations and becoming a specialist physician with the objective of recouping their investment. Under the lure of money and status, social conscience is often forgotten. This is a good introduction to Freire’s ideas. I would state, however, that the more uncomfortable aspects of social change, empowerment, social transformation and empowering the oppressed have received less emphasis both in the article and in medical education.

Reviewer Expertise:

NA

No decision status is available

# Reviewer report: Reviewer response for version 1

**DOI:** 10.21956/mep.19305.r28148

**Published:** 2017-07-05

**Authors:** Prashanti Eachempati

**Affiliations:** 1 Melaka-Manipal Medical College

This review has been migrated. The reviewer awarded 4 stars out of 5 A well written article on Freire's framework of learning, A very pertinent message that education means drawing out of both learners and educators the best of which they are capable and the mission is not to suppress or submerge identities, but to give them full opportunity to express themselves in dialogue has been shared.If the authors could highlight application of Freire's framework with some examples, it would add much more meaning to the article.

Reviewer Expertise:

NA

No decision status is available

# Reviewer report: Reviewer response for version 1

**DOI:** 10.21956/mep.19305.r28154

**Published:** 2017-07-05

**Authors:** Tan Nguyen

**Affiliations:** 1 Deakin University

This review has been migrated. The reviewer awarded 3 stars out of 5 This article provides a light overview how Paulo Freire's work can be applied in medical education. However, I feel there is other issues missing from the discussion because his work goes into greater detail on matters regarding professionalism and social justice. These key themes were absent in the paper, despite these attributes being critical towards the training and competencies of health professionals. In addition, the principles I argue would also extend beyond foundation training and applies to life-long learning post-registration through continuing professional development, and other social justice activities.

Reviewer Expertise:

NA

No decision status is available

# Reviewer report: Reviewer response for version 1

**DOI:** 10.21956/mep.19305.r28153

**Published:** 2017-07-03

**Authors:** Julie Hunt

**Affiliations:** 1 Lincoln Memorial University

This review has been migrated. The reviewer awarded 4 stars out of 5 This manuscript applies Freire's framework of learning, often accomplished as a dialogue between teacher and learner, to medical education. His theories about humanistic and progressive, freedom-based learning are well described in the manuscript. When examining how this learning theory could be applied to medical education, the conclusion is one that is quite familiar to educators: we should be promoting active learning, yet most of our content is delivered as passive learning. If anything could be added to the manuscript, it may be a discussion of how that can be accomplished, or perhaps a few examples of when or where that is well done, to help the reader take away some action points. Overall this is a good introductory article to Freire's learning framework and how it may apply to medical education broadly.

Reviewer Expertise:

NA

No decision status is available
